# Supplementary material for: The effect of mindfulness-based Tai Chi Chuan on mobile phone addiction among male college students is associated with executive functions
Source: PLoS One. 2025 May 8;20(5):e0314211. doi: 10.1371/journal.pone.0314211 (PMC12061109; doi:10.1371/journal.pone.0314211)
Supplement: S2 Data — (DOCX) [file pone.0314211.s002.docx]

**[正念太极拳干预提高男大学生抑制功能改善手机成瘾行为的作用研究](https://kns.cnki.net/kcms2/article/abstract?v=d24vdHNzaZhgCDzdZ8l_2u6l9Nd9wPst39KdJkfk8_zZJ9WitdhnusodKX9zXpr4blCkGcgXc2O-qsMYR9-xTbl34eFIqBwquk8iyj9U58J6JnLrYV_guC51ItB28QKaLfgwpmjDpw2cV5QRfRlc4Jgf7j7VKGt3HI2YFhC8Iv8OzWht6DyeB6pWoCqjBGNKDRReb2cMAJM=&uniplatform=NZKPT&language=CHS)**

**1.研究内容**

本研究以男性大学生为研究对象，探讨正念太极拳干预对认知功能和执行功能的影响，特别是抑制功能的变化与手机成瘾行为之间的关系。研究将通过8周的正念太极拳干预来比较执行功能、正念水平和手机成瘾情况的变化，从宏观层面揭示正念太极拳在改善大学生手机成瘾中的潜在作用机制。研究还将深入探讨执行功能中抑制能力的变化如何影响手机成瘾行为，从微观层面分析正念太极拳通过提升执行功能在缓解手机成瘾中的具体作用路径。通过一系列综合性的研究与分析，为正念太极拳作为干预手段提供更加系统的理论依据与实践支持。

**2.**[**正念太极拳干预提高男大学生抑制功能改善手机成瘾行为的作用研究**](https://kns.cnki.net/kcms2/article/abstract?v=d24vdHNzaZjUmlmRChL15Anv6TPfpkpZv5V-NOv4OW10IMcUzVEYrPUxflv88j1hh-3sk3x-2oM1VsFFrMCScv6m8_h1Z6Vwwp7lc34Zy4Hji35vYdilzbxuNdVEbWIUDRTkNaA3DYCW2idR-rLWMA81bXhnqzo1B6rAQrDT1AGRrhgZrVE1B7iTpydgdkQ0ml1pV7lWaw0=&uniplatform=NZKPT&language=CHS)

拟选取若干男性大学生为研究对象。纳入标准为：（1）在校非体育专业男大学生；（2）年龄≥18岁；（3）具有一定睡眠障碍，及PSQI≥6分；（4）日常学习生活中未有规律运动习惯；（5）自愿参与为期8周的正念太极拳训练，并签署知情同意书；（6）无严重疾病。

对照组不进行任何特殊运动干预，实验组接受为期8周的正念太极拳运动干预。运动干预前后分别测定受试者手机成瘾指数、正念水平、执行功能及其各项子指标。并探讨其在正念太极拳干预改善大学生手机成瘾情况中的可能机制。

- 手机成瘾指数：正念水平的测量选用的是2003年Brown和Ryan编制的正念注意觉知量表（MAAS），该量表为单维度量表，评分从1-6分，均为正向评分，共包括15道题项，总分越高代表个体有较高正念觉知和注意当下的水平。
- 正念水平：手机成瘾情况的测量采用的是手机成瘾量表（MPAI），该量表有四个维度，分别是失控性、戒断性、逃避性和低效性，评分从1分-5分，共有17个题目。
- 执行功能：测试包含Flanker任务（抑制功能）、2-back任务（刷新功能）和More-odd shifting任务（转换功能），测试指标为反应时（毫秒）和正确率（%）。
