# Supplementary material for: The effect of mindfulness-based Tai Chi Chuan on mobile phone addiction among male college students is associated with executive functions
Source: PLoS One. 2025 May 8;20(5):e0314211. doi: 10.1371/journal.pone.0314211 (PMC12061109; doi:10.1371/journal.pone.0314211)
Supplement: S3 Data — (DOCX) [file pone.0314211.s003.docx]

**Effects of Mindfulness-Based Tai Chi Chuan on Executive Functions and Mobile Phone Addiction Among Male College Students**

**1. Research Content**

This study focuses on male college students as the research subjects, exploring the impact of mindfulness-based Tai Chi intervention on cognitive function and executive function, with particular attention to the relationship between changes in inhibitory control and smartphone addiction behaviors. The study will compare changes in executive function, mindfulness levels, and smartphone addiction through an 8-week mindfulness Tai Chi intervention. On a macro level, the research aims to reveal the potential mechanisms through which mindfulness Tai Chi may improve smartphone addiction among college students. Additionally, the study will further investigate how changes in inhibitory control within executive function influence smartphone addiction behaviors, analyzing the specific pathways through which mindfulness Tai Chi alleviates smartphone addiction by enhancing executive function on a micro level. This comprehensive analysis aims to provide a more systematic theoretical basis and practical support for mindfulness Tai Chi as an intervention method.

**2.Effects of Mindfulness-Based Tai Chi Chuan on Executive Functions and Mobile Phone Addiction Among Male College Students**

A number of male college students will be selected as research subjects. The inclusion criteria are as follows: (1) male college students who are not majoring in physical education; (2) age ≥ 18 years; (3) certain sleep disturbances, with a PSQI score ≥ 6; (4) no regular exercise habits in daily life; (5) voluntary participation in an 8-week mindfulness Tai Chi training program and signing of informed consent; (6) no serious illnesses.

The control group will receive no special exercise intervention, while the experimental group will undergo an 8-week mindfulness Tai Chi intervention. Before and after the intervention, participants' smartphone addiction index, mindfulness levels, executive function, and related sub-indicators will be measured. The study will explore the possible mechanisms through which mindfulness Tai Chi intervention improves smartphone addiction among college students.

- **Smartphone Addiction Index**: The mindfulness level will be measured using the Mindful Attention Awareness Scale (MAAS) developed by Brown and Ryan in 2003. This is a unidimensional scale with scores ranging from 1 to 6, with all items positively scored. The scale consists of 15 items, with higher total scores indicating higher levels of mindfulness and attention to the present moment.
- **Smartphone Addiction**: The smartphone addiction level will be assessed using the Smartphone Addiction Proneness Scale (MPAI), which consists of four dimensions: uncontrollability, withdrawal, escapism, and inefficiency. The scale ranges from 1 to 5 and contains 17 items.
- **Executive Function**: The executive function will be assessed using tasks such as the Flanker Task (inhibitory control), the 2-back Task (working memory), and the More-odd Shifting Task (cognitive flexibility). The measures include reaction time (in milliseconds) and accuracy (percentage).
