# Supplementary material for: The effect of mindfulness-based Tai Chi Chuan on mobile phone addiction among male college students is associated with executive functions
Source: PLoS One. 2025 May 8;20(5):e0314211. doi: 10.1371/journal.pone.0314211 (PMC12061109; doi:10.1371/journal.pone.0314211)
Supplement: Attachment — Submitted filename: Response to Reviwers.docx [file pone.0314211.s006.docx]

**Response to Reviwers’**

**Answers to the academic editor**

**1.** **Please ensure that your manuscript meets PLOS ONE's style requirements, including those for file naming. The PLOS ONE style templates can be found at**

**https://journals.plos.org/plosone/s/file?id=wjVg/PLOSOne_formatting_sample_main_body.pdf and** [**https://journals.plos.org/plosone/s/file?id=**](https://journals.plos.org/plosone/s/file?id=) **ba62/PLOSOne_formatting_sample_title_authors_affiliations.pdf**

Thank you for your reminder and guidance. I will ensure that my manuscript fully adheres to the style requirements of PLOS ONE, including the conventions for file naming. I will meticulously review all pertinent formatting and naming standards to guarantee that my paper is free from any such issues. I greatly appreciate your support for my research work and look forward to the opportunity of having my paper published in PLOS ONE.

**2. PLOS requires an ORCID iD for the corresponding author in Editorial Manager on papers submitted after December 6th, 2016. Please ensure that you have an ORCID iD and that it is validated in Editorial Manager.**

My ORCID iD is 0000-0003-3930-9227 and it has been validated in Editorial Manager.

**3. Note from Emily Chenette, Editor in Chief of PLOS ONE, and Iain Hrynaszkiewicz, Director of Open Research Solutions at PLOS: Did you know that depositing data in a repository is associated with up to a 25% citation advantage (https://doi.org/10.1371/journal.pone.0230416)? If you’ve not already done so, consider depositing your raw data in a repository to ensure your work is read, appreciated and cited by the largest possible audience. You’ll also earn an Accessible Data icon on your published paper if you deposit your data in any participating repository (https://plos.org/open-science/open-data/#accessible-data).**

I've already submitted my original data files on Supporting Information of Attach Files. I'm holding off on storing the raw data in a repository, thanks for the suggestion.

**4. Thank you for stating the following financial disclosure:"The work involved in this submission was supported by the R&D Program of Beijing Municipal Education Commission(KM202210029001). The fund recipient is Baishuang, who is the corresponding author of this submission."**

**Please state what role the funders took in the study. If the funders had no role, please state: ""The funders had no role in study design, data collection and analysis, decision to publish, or preparation of the manuscript.""**

**If this statement is not correct you must amend it as needed.**

**Please include this amended Role of Funder statement in your cover letter; we will change the online submission form on your behalf.**

In our study, the financial support provided by the R&D Program of Beijing Municipal Education Commission (KM202210029001) was pivotal in facilitating the research. However, I confirm that the funders had no role in the study design, data collection and analysis, decision to publish, or preparation of the manuscript. Their contribution was strictly financial and did not extend to any involvement in the scientific aspects of the study.

Therefore, the Role of Funder statement for our submission will be: "The funders had no role in study design, data collection and analysis, decision to publish, or preparation of the manuscript."

I will include this amended statement in the cover letter of our submission. I appreciate your assistance in modifying the online submission form accordingly and ensuring the transparency and integrity of our research disclosure.

**5. Please include your full** **ethics statement in the ‘Methods’ section of your manuscript file. In your statement, please include the full name of the IRB or ethics committee who approved or waived your study, as well as whether or not you obtained informed written or verbal consent. If consent was waived for your study, please include this information in your statement as well.**

Thank you for your valuable feedback. In response to your request, I will include a comprehensive ethics statement and the fact that participants have been provided with written information in the 'Methods' section of my manuscript. I will make these revisions promptly and resubmit the manuscript accordingly.

**6. Please review your reference list to ensure that it is complete and correct. If you have cited papers that have been retracted, please include the rationale for doing so in the manuscript text, or remove these references and replace them with relevant current references. Any changes to the reference list should be mentioned in the rebuttal letter that accompanies your revised manuscript. If you need to cite a retracted article, indicate the article’s retracted status in the References list and also include a citation and full reference for the retraction notice.**

I have completely revised the formatting of the references in accordance with the requirements of PLOS ONE and have confirmed that the current references do not use the retracted articles.

In addition, I have added four pieces of literature. Two^[10,28]^ of them were added as updated literature based on reviewer 2's comments, one^[21]^ was added with charts and corresponding literature based on reviewer 2's comments, and one^[31]^ was added with references to exercise intervention programs based on reviewer 1's comments.

*10.	Liu F, Xi Y, Li N, Wu M. Brief Mindfulness Training Mitigates College Students' Mobile Phone Addiction: The Mediating Effect of the Sense of Meaning in Life. Psychol Res Behav Manag. 2024;17:273-82. https://doi.org/10.2147/PRBM.S439360*

*28.	Liebherr M, Brandtner A, Brand M, Tang YY. Digital mindfulness training and cognitive functions: A preregistered systematic review of neuropsychological findings. Ann N Y Acad Sci. 2024;1532(1):37-49. https://doi.org/10.1111/nyas.15095*

*21.	Riglin L, Collishaw S, Richards A, Thapar AK, Maughan B, O'Donovan MC, et al. Schizophrenia polygenic risk score and psychotic risk detection-Authors' reply. Lancet Psychiatry. 2017;4(3):188-9. https://doi.org/10.1016/S2215-0366(17)30052-4*

*31. Zheng G, Lan X, Li M, Ling K, Lin H, Chen L, et al. The effectiveness of Tai Chi on the physical and psychological well-being of college students: a study protocol for a randomized controlled trial. Trials. 2014;15:129. https://doi.org/10.1186/1745-6215-15-129*

**Answers to reviewer 1**

**1. Random allocation is mentioned in the research methods section of this paper. Please clarify the specific method of random allocation. Please describe the process of assigning concealment.**

Thank you for your inquiry regarding the specifics of the random allocation process employed in our research. To clarify, we utilized a computer-generated randomization sequence to assign participants to either the intervention group practicing mindfulness-based Tai Chi or the control group. This randomization ensured an unbiased allocation and equal probability for each participant to be assigned to either group.

Regarding the concealment of assignment, we implemented a secure, opaque, and sealed envelope system. Each envelope contained the group assignment and was only opened after the enrolled participant completed all baseline assessments. This method was chosen to prevent any selection bias and to maintain the integrity of the random allocation. By employing these rigorous methods, we aimed to enhance the validity and reliability of our study's outcomes.

I will make sure to include these details in the revised manuscript to provide a comprehensive understanding of our methods. Thank you for highlighting the importance of transparency in describing the randomization process.

**2. The exercise dose in the exercise intervention Plan section proposes, “The exercise time lasts 40 min each time, including 5 min of wuji pile practice with positive thoughts, 30 min of eight styles of Tai Chi Chuan practice (4 min*6 sets, 1 min interval between sets), and 5 min of relaxation part of wuji pile with music. The exercise frequency is 3 times a week. "What is it based on, Please support this in the Introduction section.**

Thank you for your insightful query regarding the basis for the exercise dose outlined in our intervention plan. In response, I will provide the necessary support and rationale in the Methods section of the revised manuscript.

The decision for a 40-minute exercise duration, comprising Tai Chi Chuan and wuji pile practices, is based on previous research^[31]^ indicating the effectiveness of moderate-duration Tai Chi exercises in enhancing cognitive and mental health. The inclusion of the cited studies in the bibliography provides a clearer understanding of the design of exercise interventions and their basis in the existing literature and practice guidelines.

*31. Zheng G, Lan X, Li M, Ling K, Lin H, Chen L, et al. The effectiveness of Tai Chi on the physical and psychological well-being of college students: a study protocol for a randomized controlled trial. Trials. 2014;15:129. https://doi.org/10.1186/1745-6215-15-129*

**Answers to reviewer 2**

**1. It is suggested to add biological charts or literature in the introduction and discussion section to better support the argument.**

Thank you for your suggestion to enhance the manuscript by including biological charts and relevant literature in the Introduction and Discussion sections. This addition will undoubtedly strengthen the argument and provide a more comprehensive understanding of the context and implications of the study.

In the Introduction, I will incorporate biological charts that visually represent the neurological and physiological mechanisms underlying the impact of Executive functioning related to the reward effect of addictive behaviors^[21].^ I will proceed with these revisions promptly and ensure they are seamlessly integrated into the manuscript.

*21.	Riglin L, Collishaw S, Richards A, Thapar AK, Maughan B, O'Donovan MC, et al. Schizophrenia polygenic risk score and psychotic risk detection-Authors' reply. Lancet Psychiatry. 2017;4(3):188-9. https://doi.org/10.1016/S2215-0366(17)30052-4*

**2. There are a lot of recent studies on mindfulness, executive function and mobile phone addiction, so you can refer to recent literatures to provide theoretical basis for this study.**

Thank you for your valuable suggestions regarding the inclusion of recent literature on positive thinking, executive functioning, and cell phone addiction. I appreciate you emphasizing the importance of grounding our research in current research and theoretical developments. In response to your feedback, I will include in the manuscript the most recent research exploring the relationship and implications between positive thinking exercises, executive functioning, and cell phone addiction, including references 10. and 28.

*10.	Liu F, Xi Y, Li N, Wu M. Brief Mindfulness Training Mitigates College Students' Mobile Phone Addiction: The Mediating Effect of the Sense of Meaning in Life. Psychol Res Behav Manag. 2024;17:273-82. https://doi.org/10.2147/PRBM.S439360*

*28.	Gao L, Zhang J, Xie H, Nie Y, Zhao Q, Zhou Z. Effect of the mobile phone-related background on inhibitory control of problematic mobile phone use: An event-related potentials study. Addict Behav. 2020;108:106363. https://doi.org/10.1016/j.addbeh.2020.106363*

**Answers to reviewer 3**

1. **- editing the title appropriately.**

Thank you for your recommendation to edit the title of the manuscript for better suitability. Therefore, I agree that refining the title as “The effect of Mindfulness-Based Tai Chi Chuan on Mobile Phone Addiction Among Male College Students is associated with Executive Functions.”

**2. - use of table descriptions, in accordance with the editorial guidelines - description above the table and figures below the table.**

Thank you for pointing out the need to follow editorial guidelines for the placement of instructions for tables and figures in manuscripts.

I will check the manuscript to ensure that each table and figure is accompanied by an explanation in the correct place according to editorial standards.

**3. standardize the notation for presenting the significance of "p" or "P". Mediating effects test method - Statistical analyzes were all performed on SPSS 23.0 with a statistical analysis significance of p<0.05. The following text uses the notation "P"**

Thank you for drawing attention to the inconsistency in the notation of statistical significance in our manuscript. I understand the importance of standardizing such notations for clarity and accuracy in scientific communication.

In response to your observation, I will ensure uniformity in the notation for presenting statistical significance throughout the manuscript. As it is a common convention in scientific literature to denote probability values with a lowercase "p," I will adhere to this standard. Hence, all references to statistical significance will consistently use "p" rather than alternating between "p" and "P.

I appreciate your attention to this detail, which is crucial for maintaining the rigor and precision of our research presentation. I will proceed to make these revisions immediately for the resubmitted version of the manuscript.

**4. - you should consider presenting the results for both groups in a descriptive form (providing the data that is in the tables), especially in the context of the control group:**

**“There were no significant differences between pre- and post-test correct speed (F = 0.113, P = 0.738 > 0.05) and updating reaction time (F = 0.112, P = 0.739 > 0.05) in the control group.”**

However, after the textual description, I have already visualized the results in a bar chart, so I don't think it is necessary to add a table to show the data.

**5. Conclusion 1 and 3 are basically the same, just composed differently. They constitute a summary of the research conducted rather than conclusions. I would suggest expanding:**

**MTCC exercises significantly increase cognitive functions, leading to increased inhibition and attentiveness, and may be helpful in the prevention of addictions, including cell phone addictions.**

Thank you for your insightful feedback regarding the conclusions of our study. In line with your suggestion, I will revise and consolidate these conclusions to clearly articulate the specific impacts of Mindfulness-Based Tai Chi Chuan (MTCC) exercises. I will emphasize how our study demonstrates that MTCC exercises significantly enhance cognitive functions, particularly in terms of increased inhibitory control and attentiveness. This will be expanded to illustrate the potential application of these exercises in the prevention of addictions, with a special focus on cell phone addiction. I will ensure these changes are reflected in the revised manuscript.

**6. Lack of properly compiled literature, multiple omission of journal titles presented in the literature list: items: 2,3,4,6, 7 (item from PLOS ONE), 8,12,14 etc.**

**Example position 20**

**There is: Diamond AJArop. executive functions. 2013,64:135-68.**

**It should be: Diamond A. Executive functions. Annu Rev Psychol. 2013; 64: 135-68. doi: 10.1146/annurev-psych-113011-143750**

In response to your comments, I will thoroughly check the bibliography to ensure that all journal titles for each reference item are listed correctly and completely. The issues you have pointed out will be carefully addressed. I will cross-reference each citation with the corresponding source to verify the accuracy and completeness of the journal name.
